# Effect of purified omega-3 fatty acids on reducing left ventricular remodeling after acute myocardial infarction (OMEGA-REMODEL study): a double-blind randomized clinical trial)

**DOI:** 10.1186/1532-429X-17-S1-O7

**Published:** 2015-02-03

**Authors:** Bobby Heydari, Siddique A Abbasi, Ravi Shah, Shuaib Abdullah, Jiazuo Feng, William Harris, Joe McConnell, Evan Appelbaum, Udo Hoffmann, Michael Steigner, Ron Blankstein, Elliott A Antman, Michael Jerosch-Herold, Raymond Y Kwong

**Affiliations:** 1 Cardiology, University of Calgary, Calgary, AB, Canada; 2 Cardiology, Brigham and Women's Hospital, Boston, MA, USA; 3 Radiology, Massachusetts General Hospital, Boston, MA, USA; 4 Cardiology, BIDMC, Boston, MA, USA; 5 Health Diagnostic Laboratory, Richmond, VA, USA; 6 Cardiology, UT Southwestern, Dallas, TX, USA

## Background

Acute myocardial infarction (MI) remains a leading cause of patient death and morbidity. Prognosis following MI has been shown to be strongly associated with small changes in left ventricular remodeling. Omega-3 fatty acid (PUFAs) supplementation may have a number of beneficial pleiotropic effects, including enhancement of myocardial relaxation^1^ and reduction of vascular tone.^2^ One prospective, randomized trial demonstrated significant survival benefit for PUFAs following acute MI.^3^ However, the mechanism and potential myocardial changes during the convalescent phase post MI from PUFAs have not been well described.

## Methods

The OMEGA-REMODEL study was a randomized, double-blinded, placebo controlled trial of PUFA supplementation post acute MI. A total of 358 patients were randomized to study therapy with PUFAs (n=180) or matching placebo (n=178) and underwent baseline assessment by CMR 4-28 days following MI, with follow-up after 6 months of randomized therapy. The primary endpoint was changes in left ventricular end-systolic volume indexed to body surface area (LVESVI). Secondary outcomes included a) change in total infarct size, b) expansion of MECVF within noninfarcted myocardium, and c) changes in systemic biomarkers.

## Results

**Figure 1:**
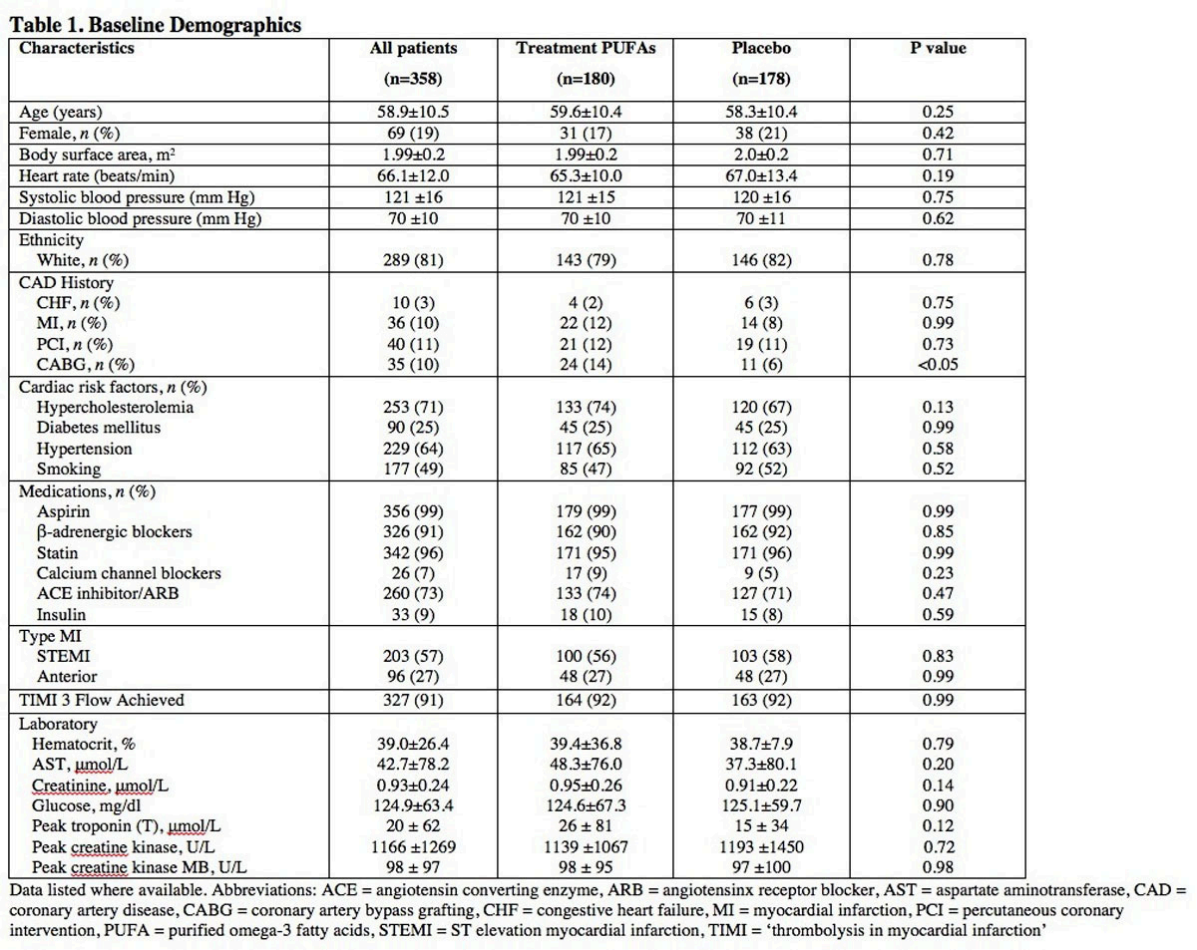
Baseline demographics

Baseline demographics for the entire cohort and both treatment arms are shown in Table 1. CMR characteristics, PUFAs levels, and biomarkers for baseline and 6-month post-treatment values of each treatment arm are shown in Table 2 and Figure 1. There were no significant differences for any ventricular parameters, infarct size or MECVF at baseline. After 6 months of therapy, follow-up CMR revealed a significant difference for percent change of LVESVI (-5.4±16.6 PUFAs versus 0.75±21.0 Placebo, p=0.01) and MECVF (-1.3±14.9 PUFAs versus 3.8±18.0 Placebo, p=<0.05) between the treatment arms. In addition, the inflammatory biomarkers, hsCRP and myeloperoxidase, were substantially reduced in the PUFAs treated arm as compared with the placebo arm (-98.2±243.6% versus -30.5±221.5% p<0.01, and -0.8±6.7 versus +0.8±5.3 p=<0.05, respectively). Finally, the percent reduction in ST2 after 6 months, a biomarker of myocardial fibrosis, was significantly greater in the PUFAs treated arm (-1.35±8.0 versus +0.92±7.5, p=0.03). Adjusted multivariable analysis demonstrated an independent association for change in PUFAs levels with adverse LV remodeling.

**Figure 2:**
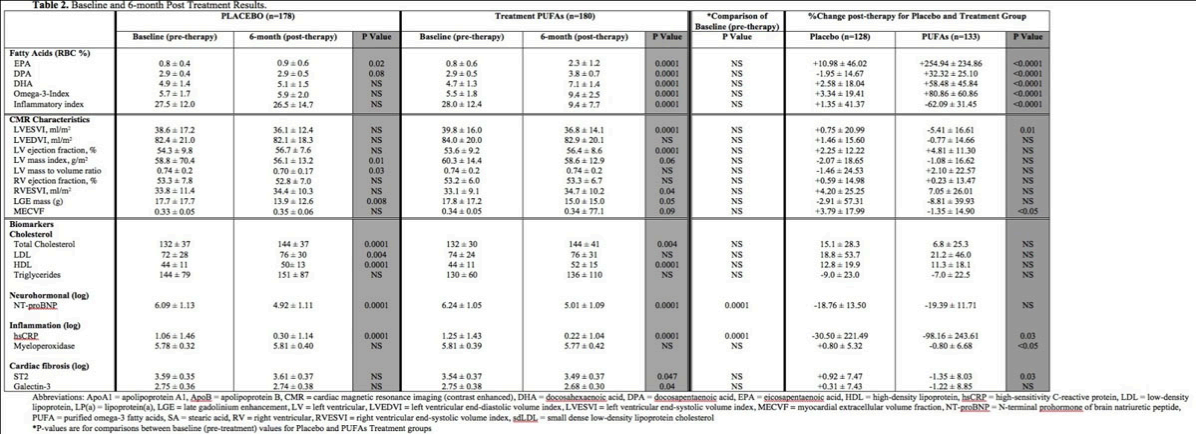
Baseline and 6-month post treatment results

## Conclusions

The results of this study demonstrated a significant reduction in LVESVI and MECVF for patients treated with high-dose PUFAs as compared to placebo. Further assessment of systemic biomarkers demonstrated a substantial reduction in biomarkers of inflammation, and fibrosis for the PUFAs treated arm. These findings represent the first description of the effect of PUFAs on myocardial tissue phenotypes during the convalescent phase post MI and may suggest potentially important pathophysiological pathways for their pleiotropic effects.

## Funding

National Institutes of Health Heart, Lung and Blood Institute (NHLBI).

